# *S. aureus* Colonization, Biofilm Production, and Phage Susceptibility in Peritoneal Dialysis Patients

**DOI:** 10.3390/antibiotics9090582

**Published:** 2020-09-07

**Authors:** Karlis Racenis, Juta Kroica, Dace Rezevska, Lauris Avotins, Edgars Skuditis, Anna Popova, Ilze Puide, Viktorija Kuzema, Aivars Petersons

**Affiliations:** 1 Department of Biology and Microbiology, Riga Stradins University, LV-1007 Riga, Latvia; juta.kroica@rsu.lv (J.K.); dace.rezevska@rsu.lv (D.R.); lauris.avotins@rsu.lv (L.A.); e.skuditis@gmail.com (E.S.); annasild@yahoo.co.uk (A.P.); 2 Center of Nephrology, Pauls Stradins Clinical University Hospital, LV-1002 Riga, Latvia; Ilze.Puide@rsu.lv (I.P.); Viktorija.Kuzema@rsu.lv (V.K.); Aivars.Petersons@rsu.lv (A.P.); 3 Department of Internal Diseases, Riga Stradins University, LV-1007 Riga, Latvia

**Keywords:** *S. aureus*, peritoneal dialysis, phage adaptation, biofilm, phage therapy, phenotypic trait

## Abstract

Peritonitis caused by *Staphylococcus* *aureus* is of major importance in peritoneal dialysis (PD) patients due to its great virulence profile and biofilm formation ability. Bacteriophages are a potential tool to treat peritonitis resulting from biofilm-associated infections. We screened *S. aureus* colonization in 71 PD patients from the nasal cavity, groin, and PD exit-site regions and analyzed clinical outcomes in these patients. We performed biofilm-formation testing of different strains and compared the isolates of one patient to detect phenotypic differences in *S. aureus*. Phage cocktails were used to detect *S. aureus* in vitro susceptibility. An adaptation procedure was performed in cases of bacterial resistance. Around 30% of PD patients (*n* = 21) were found to be *S. aureus* carriers; from these, a total of 34 *S. aureus* strains were isolated, of which 61.8% (*n* = 21) produced a strong biofilm. Phenotypic differences in strain biofilm production were detected in eight patients out of ten. All strains were sensitive to commonly used antibiotics. Broadly positive phage lytic activity (100%) was observed in six cocktails out of seven, and bacterial resistance towards phages was overcome using adaptation. Overall phages showed a promising in vitro effect in biofilm-forming *S. aureus* strains.

## 1. Introduction

*Staphylococcus aureus* is a common causative agent of bacterial peritonitis in patients undergoing peritoneal dialysis (PD), representing 6–14% of cases [1,2,3], as well as being the most common causative agent of exit-site infection [4,5]. *S. aureus* peritonitis is associated with higher rates of relapse, repeated infection, and catheter removal in addition to increased mortality [3,6]. This can be explained by the existence of particular strains that have high expression of virulence factors such as Panton–Valentine leukocidin, enterotoxins that can act as superantigens, hemolysins, phenol-soluble modulins, and the quorum-sensing accessory gene regulator (*agr*) system that plays an important role in biofilm formation and virulence factor expression [7,8]. *S. aureus* colonizes human organisms in various sites such as the nasal cavity, inguinal region, and the PD catheter exit site [5]; therefore, treatment of culture-positive nasal carriers with topical mupirocin and the application of antibiotics to the catheter exit-site are recommended to reduce the risk of peritonitis [9]. Nevertheless, bacterial biofilm formation is of great importance as *S. aureus* can produce multilayered biofilms that are mainly made of polysaccharides [10]. These structures evade both the innate and adaptive immune systems of the host, leading to altered polymorphonuclear neutrophil killing and weakened phagocytosis, ineffectiveness of toll-like-receptor activation [8,11], and activation of the T-helper 1 (Th1) and Th17 immune response instead of the Th2 response that prevents *S. aureus* biofilm-associated infection [12]. The antimicrobial effect within the biofilm is insufficient due to lowered bacterial metabolic activity and reduced penetration that can be observed even with such bactericidal antimicrobials as oxacillin and vancomycin [13,14]. In a Brazilian study, biofilm formation was detected in 89% of *S. aureus* strains that were isolated from patients with PD-associated peritonitis [15]. Different anatomical niches of the xhuman organism can be colonized by phenotypically and genotypically polyclonal *S. aureus* strains; even the nasal cavity can be colonized with different *S. aureus* strains serving as a source for horizontal gene transfer with the ability for diverse antimicrobial susceptibility and virulence factor expression [16,17]. The quorum-sensing accessory gene regulator (*agr*) system plays a role in biofilm formation, and low *agr* activity is associated with increased biofilm production [18]; however, their expression activity is linked to environmental conditions [19]. Detection of strain polyclonality revealed differences in *agr* gene types [17], and the *S. aureus* biofilm-formation capacity varies between isolates and according to *agr* type [20]. Bacterial isolation sites among clinical strains impact biofilm production in *S. aureus* infections [21].

Bacteriophages or phages are natural viruses that infect bacterial cells and replicate in them, causing bacterial cell lysis. Eventually, this property can be used to eradicate bacterial pathogens. Phages are very diverse due to differences in their size, genetic structure, and morphology. Moreover, the host range of individual phage may differ from strain-specific to the extent of infecting bacteria that derive from distinctive genera [22]. Bacterial host specificity of phages is regulated via host cell surface receptors and, hence, bacteria that do not expose a specific receptor for a particular bacteriophage cannot be infected [23]. Phages replicate at the site of infection and produce substances such as endolysins that act on bacterial cell wall peptidoglycan, polysaccharide depolymerases that degrade carbohydrates, and lipopolysaccharides within bacterial biofilm. This leads to the destruction of biofilm and the bacteria embedded within regardless of their various metabolic activities. Phage-produced enzymes also mainly act on single bacterial species [24,25]. Such properties make bacteriophages and their produced particles an attractive tool for *S. aureus* eradication and the treatment of peritonitis caused by both antibiotic-resistant and -sensitive strains [26,27,28].

In this study, we detected *S. aureus* colonization in PD patients in the nasal cavity, groin, and PD exit-site region. We analyzed the impact of *S. aureus* colonization on peritonitis, death, kidney transplantation and PD catheter removal rates. Commercially available phage cocktails that may be potentially potent in future clinical trials were used to detect *S. aureus* in vitro susceptibility. The adaptation procedure was performed in the case of bacterial resistance towards phages. We performed biofilm-formation testing of different strains and compared the isolates of one patient to detect *S. aureus* phenotypic differences.

## 2. Materials and Methods

We included all peritoneal dialysis patients in Pauls Stradins Clinical University Hospital Peritoneal Dialysis Department, Latvia, between November 2016 and January 2017. *S. aureus* isolation and in vitro testing of antimicrobial and phage susceptibility, phage adaptation and strain biofilm formation testing were performed. Underlying diseases were noted for each patient and clinical outcomes as peritonitis, death, removal of PD catheter, and transplantation were analyzed over two-year period after *S. aureus* screening.

### 2.1. S. aureus Isolation and Microbiological Investigation

Swabs were obtained from nasal, inguinal, and PD catheter sites. Material was transported with universal transport media (AMIES) at room temperature within 2 h. Microbiological analysis was performed after overnight swab culturing on trypticase soy broth, mannitol salt agar, and Baird Parker agar with egg yolk tellurite. Gram staining and microscopy, coagulase detection, catalase test, and latex agglutination (Oxoid) were performed. For the final identification of *S. aureus*, a VITEK-2 (bioMerieux) system was used. Disk-diffusion antimicrobial susceptibility testing was performed according to EUCAST version 7.1, 2017.

### 2.2. Biofilm Growth Using Microtiter Plate Assay

Bacterial strains were isolated on trypticase soy agar (TSA) by streak method with incubation at 37 °C for 16–18 h. Two to three colonies were taken and resuspended in trypticase soy broth (TSB) supplemented with an additional 1% glucose for incubation at 37 °C for 16–18 h to receive a concentration 1–3 × 10^9^ CFU/mL, bacterial concentration was detected using densitometer (DEN-1, Riga, Latvia) occasionally to reaffirm bacterial concentration we would perform bacterial titration. Inoculum bacteria grown in broth were diluted 1:100 in TSB supplemented with 1% glucose to receive a final concentration 1–3 × 10^7^ CFU/mL. Then, 150 µL of diluted suspension was transferred with multichannel pipette in sterile 96-well plate (96-well TC plate, Suspension, F, Sarstedt, Germany) for biofilm cultivation. Each plate contained 11 strains and the negative control, with 8 wells per strain to provide reliable analysis of the experiment, which was triplicated. The inoculated plate was cultivated aerobically at 37 °C for 24 h. After incubation, the content of the plate was carefully removed by decanting in disinfectant. Each well was rinsed three times with 250 µL 0.9% saline to remove planktonic bacteria. After washing, the remaining bacteria attached were heat-fixed for 60 min at 60 °C. Staining was performed adding 150 µL of 0.1% crystal violet per well over 15 min; then, color was removed by decanting, and each well was washed three times with 250 µL distilled water. To detach formed cells from the wells and quantify the biofilm production degree, 150 µL of 96% ethanol was added to each well and left for 30 min (see Figure S1). Afterwards, the optical density (OD) of wells was measured with a microplate spectrophotometer (TECAN INFINITE F50, Männedorf, Switzerland) at 570 nm wavelength. Data were recovered from the TECAN interface and transferred to Microsoft Excel 10.

### 2.3. Biofilm Calculation

The optical density (OD) values for each strain were averaged and expressed as a number. The cut-off value (ODc) was separately calculated as three standard deviations (SDs) over the mean negative control value for each plate. To classify bacterial strains according to their capability for biofilm production, they were grouped according to Stepanovic et al. [29], respectively, biofilm nonproducer ODs ≤ ODc, weak producer ODc < ODs ≤ 2 × ODc, moderate producer 2 × ODc < ODs ≤ 4 × ODc, and strong producer ODs ≥ 4 × ODc.

### 2.4. Bacteriophage Preparation

Seven commercial bacteriophage stocks, i.e., cocktails were used: 6 produced by Eliava BioPreparations Ltd. (Staphylococcal, Pyo, Ses, Fersisi, Enko, and Intesti; Tbilisi, Georgiaand 1 by Microgen Ltd. (Pyobacteriophag; Perm, Perm Territory, Russia). *S. aureus* ATCC 4336 and ATCC 15923 were used as reference strains.

In terms of commercially produced phage stocks, main constituents, and host range of them have been disclosed before [30,31,32,33,34,35,36,37,38]. Staphylococcal bacteriophage containing Sb-1 (*Myoviridae*), active against *S. aureus* [30,31]. Pyo bacteriophage containing Sb-1 (*Myoviridae*) and ISP (*Myoviridae*) active against *Staphylococcus* spp., *Streptococcus* spp., *Proteus* spp., *P. aeruginosa*, and *E. coli* [32,33]. Ses active against *Staphylococcus* spp., *Streptococcus* spp., enteropathogenic *E. coli* [34]. Fersisi containing B1 (*Myoviridae*) and JA1 (*Myoviridae*) active against *Staphylococcus* spp., *Streptococcus* spp. [35,36]. Enko active against *Salmonella* spp., *Shigella* spp., *Staphylococcus* spp., enteropathogenic *E. coli* [34]. Intesti containing D1-D18, F1-F4 and Proteus phage (*Myoviridae*, *Siphoviridae*, *Podoviridae*) [37], active against *Shigella* spp., *Salmonella* spp., *E.coli*, *Proteus* spp., *Staphylococcus* spp., *P.aeruginosa*, *Enterococcus* spp. [32,34]. Pyobacteriophag containing fRuSau02 (*Myoviridae*) and SCH1 *(Podoviridae)* active against *Staphylococcus* spp., *Streptococcus* spp., *Enterococcus* spp., *Proteus* spp., *Klebsiella* spp., *P. aeruginosa*, and *E.coli* [33,36,38].

Plaque assay was used for the determination of the original titer of all 7 commercial phage cocktails that was measured in plaque-forming units per milliliter (PFU/mL). Serial tenfold dilutions of each commercial bacteriophage cocktail were made in 1.5 mL microcentrifuge tubes labeled from 10^−1^ to 10^−5^. For sequential dilutions, each microcentrifuge tube was filled with 900 µL sterile physiological saline and 100 µL bacteriophage suspension. Two to three pure colonies of *S. aureus* ATCC 4336 bacterial strain freshly grown on TSA plate were transferred to Luria–Bertani (LB) broth medium and incubated overnight at 37 °C. Then, 100 µL of bacterial culture suspension grown overnight and 50 µL of each previously made dilution of every bacteriophage cocktail were mixed together in 4 mL of previously molten 0.7% TSA tube, gently mixed, and poured evenly onto each corresponding TSA plate. After solidification of the top agar, plates were inverted and incubated overnight at 37 °C. Each bacteriophage commercial stock titer (PFU/mL) was calculated by using the following equation: (number of plaques)/(dilution factor × volume of diluted bacteriophage in mL). All procedures were performed in duplicate.

To attain the higher concentration of each bacteriophage, cocktail phage propagation using *S. aureus* ATCC 4336 was performed. After performing the plaque assay, the webbed plates of each bacteriophage cocktail were selected. Then, 12 mL of the LB broth medium was poured on each webbed plate. The flooded plates were left at room temperature on an orbital shaker (50 rpm) for 1–2 h. Subsequently, supernatant and soft overlay agar were collected for each bacteriophage cocktail in a labeled 15 mL plastic centrifuge tube. For each labeled tube, chloroform (CHCl_3_) with a final volume of 2–3% was added. After brief vortexing, all tubes were stored for 1–2 h at 4 °C. Tubes were centrifuged at 6000× *g* for 15 min at 4 °C to remove bacterial cell debris. To avoid bacterial contamination, the bacteriophage supernatant after centrifugation was filtered using a 0.20 µm filter. The final titer of the freshly acquired bacteriophage lysate was assessed via plaque assay. To equate the dilution factor among each bacteriophage cocktail, dilution with LB broth medium was accordingly performed.

### 2.5. Bacteriophage Adaptation

The adaptation procedure was applied to overcome bacteriophage resistance and ensure the enhanced bacteriophage lytic efficacy of bacteriophage cocktails. For bacteriophage adaptation, a protocol of a modified Appelman’s method was followed [39]. To optimize the method, strain ATCC 15923 was adapted using all 7 bacteriophage cocktails. Briefly, consecutive serial tenfold dilutions were made by adding 0.5 mL bacteriophage cocktail to 4.5 mL LB broth using bacteriophage lysate that was attained after the propagation procedure. For each tube except the first, 50 µL of the overnight bacterial host was added. The first tube, labeled as 10^−1^ containing only bacteriophage cocktail, was used as negative control, whereas the tube containing only bacterial suspension was the positive control. All prepared tubes were incubated for 48 h at 37 °C. Tubes were subsequently examined by measuring OD at 600 nm. The tube with the highest dilution factor but with OD similar to negative control was selected for further steps of the adaptation procedure. Prior to centrifugation, chloroform (CHCl_3_) with a final concentration of 2–3% was added to the selected bacteriophage and bacterial host mixture and incubated at 4 °C for 2 h. Centrifugation for 15 min at 4 °C was performed at 6000× *g* to pellet bacterial debris. The bacteriophage lysate from the supernatant above the solid residue was filtered through a 0.20 µm filter. The acquired lysate was subject to 3 cycles of the described adaptation procedure before performing a spot test to visually demonstrate the lytic ability of adapted bacteriophage stock. 

### 2.6. Bacteriophage Lytic Activity Detection

To qualitatively demonstrate the lytic activity of the original and adapted bacteriophages, a spot test was applied. To make a bacterial lawn, 100 µL of overnight bacterial culture was mixed with 4.5 mL of previously molten top 0.7% TSA in a tube and gently mixed. Afterwards, it was transferred to the corresponding stiffened TSA plate and left at room temperature for approximately 20 min until complete solidification. A drop containing 10 µL of bacteriophage lysate was pipetted onto previously labeled area on the bacterial host lawn. Plates were left at room temperature to allow the drops to absorb into the agar. Plates were incubated in inverted position overnight at 37 °C. After incubation, the zones of clearing were visually examined for each plate. A positive lytic effect was recorded as confluent lysis (+++), partial lysis (++), or individual plaques (+), while a negative effect as resistant or no lysis (−) (see Figure 2). To validate the results, the spot test procedure duplicated for each phage preparation.

### 2.7. Data Analysis

IBM SPSS Statistics version 26 and Microsoft Excel 10 were used for data analysis. Graphical analysis was done using GraphPad Prism software version 8.1.0.

### 2.8. Ethical Statement

The study protocol was approved by the Ethics Committee of Riga Stradins University (document no. 32/28.01.2016). Written informed consent was obtained from all subjects before the study. 

## 3. Results

Seventy-one patients were screened for *S. aureus* carriage, but seventy patients were included in the study, as one patient with PD catheter had not initiated dialysis; 51% (*n* = 36) were male and 49% (*n* = 34) were female, with an average age 59.96 years (SD15.9). For detailed patient data see Table S1.

Causes for end-stage renal disease vary in the study group: glomerulonephritis (GN; 40%, *n* = 28), diabetic nephropathy (14.3%, *n* = 10), chronic interstitial nephritis (20%, *n* = 14), autosomal dominant polycystic kidney disease (ADPKD; 10%, *n* = 7), hypertensive nephropathy (12.9%, *n* = 9); and unknown (2.9%, *n* = 2).

*S. aureus* carriers were 30% (*n* = 21; see Table 1); 71.4% of carriers were male (*n* = 15) and 28.6% female (*n* = 6). Of the carrier group patients, 28.6% had diabetes mellitus (*n* = 6), compared to only 10.2% in the noncarrier group (*n* = 5). Prior to and during the study, none of the patients used topical antimicrobial for *S. aureus* decolonization. The mean length of patient participation in the study in the noncarrier group was 16.61 months, and 13.95 months in the carrier group.

In total, 34 *S. aureus* strains were obtained from 213 patient samples, all sensitive to commonly used antibiotics (cefoxitin, ciprofloxacin, trimethoprim/sulfamethoxazole, clindamycin, gentamycin, tetracycline, rifampicin), two strains were resistant to erythromycin. None of the isolated strains were methicillin-resistant *S. aureus* (MRSA) (see Table S2). No statistically significant correlation was noted between *S. aureus* carriage and comorbidities such as diabetes mellitus (*p* = 0.05), chronic heart failure, viral hepatitis, gout, and chronic obstructive pulmonary disease.

Death as an outcome was detected in 31.1% (*n* = 8) of carriers and 16.3% (*n* = 8) in the noncarrier group. Data showed the statistical tendency that risk of death in the carrier group was 2.33 times greater than that in the noncarrier group (see Table 2).

We noted 32 cases of peritonitis during our study (78.1%, *n* = 25 in noncarrier group; 21.9%, *n* = 7 in carrier group). Causative agents in the carrier group were mixed culture of methicillin-sensitive *S. aureus* (MSSA)/*Pseudomonas* spp. (14.3%, *n* = 1), *Streptococcus* spp. (42.9%, *n* = 3), and culture-negative (42.9%, *n* = 3). In the noncarrier group, causative agents were *Streptococcus* spp. (36%, *n* = 9), culture-negative (28%, *n* = 7), MSSA (8%, *n* = 2), mixed culture of *Aerococcus* spp./*Pseudomonas* spp., *Bacillus* spp., *Candida* spp., *Enterococcus* spp., *Aeromonas* spp., methicillin-sensitive coagulase-negative *Staphylococcus* and methicillin-resistant coagulase-negative *Staphylococcus*, each 4%, *n* = 1.

The overall incidence of peritonitis was 0.35 episodes per patient year. In the noncarrier group, there were 0.37 episodes per patient year compared to 0.29 per patient year in carrier group.

### 3.1. Biofilm Growth

**Figure 1:**
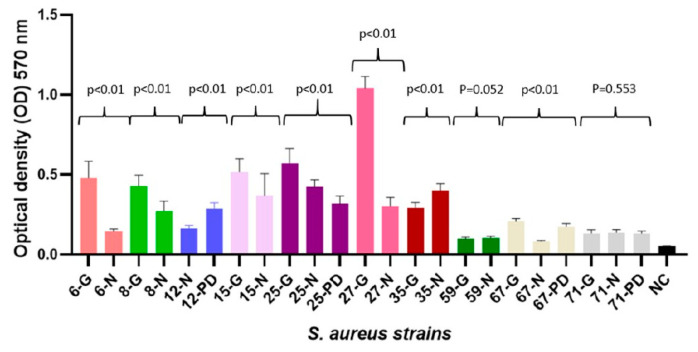
Biofilm production capability on microtiter plate of the 34 clinical isolates of *S. aureus* and negative control (NC). Bars represent mean values of OD (measured at wavelength of 570 nm). Differences between one suspect *S. aureus* strain capability in biofilm formation were analyzed using T test and Mann–Whitney U Test for two strain analyses, and one-way ANOVA and Kruskal–Wallis test for three strain analysis and are expressed as *p*-values.

Biofilm production was observed among all isolated strains. Most commonly, strains produced strong biofilm (21, 61.8%), moderate (10, 29.4%), and weak (3, 8.8%) biofilm, all isolated strain biofilm mean optical densities are summarized in Table S2. Strong biofilm production of *S. aureus* strains was detected in seven out of ten individuals who did carry *S. aureus* in multiple isolation sites (see Table 1). The biofilm production capacity of *S. aureus* strains significantly varied in 8 out of 10 patients when strains were compared among different patient *S. aureus* isolation sites (see Figure 1).

### 3.2. Bacteriophage Susceptibility and Adaptation

From all seven commercial bacteriophage cocktails, the Staphylococcal Bacteriophage (Eliava) had an original titer of 10^4^ PFU/mL, while Pyo, Enko, Intesti Bacteriophages (Eliava) and Pyobacteriophag (Microgen) had 10^5^ PFU/mL. Only the Ses and Fersisi Bacteriophages (Eliava) demonstrated a titer of 10^6^ PFU/mL. After bacteriophage cocktail propagation, of seven phage stocks all but one presented a titer of 10^9^ PFU/mL, namely Pyo, Ses, Fersisi, Intesti and Pyobacteriophag. Conversely, an estimated titer of Staphylococcal Bacteriophage was 10^7^ PFU/mL. All phage titers were equalised to 10^7^ PFU/mL before phage lytic activity testing against *S. aureus* strains (see Table 3).

The evaluation results of bacteriophage lysate lytic activity obtained in the spot assay are shown in Table 3. When tested against all 34 *S. aureus* isolates, 6 bacteriophage stocks except Staphylococcal Bacteriophage (Eliava) revealed positive lytic results in all cases. Bacterial resistance to bacteriophage represented in the spot test was determined in 9 (26%) out of 34 *Staphylococcus aureus* isolates to Staphylococcal bacteriophage (Eliava).

**Figure 2:**
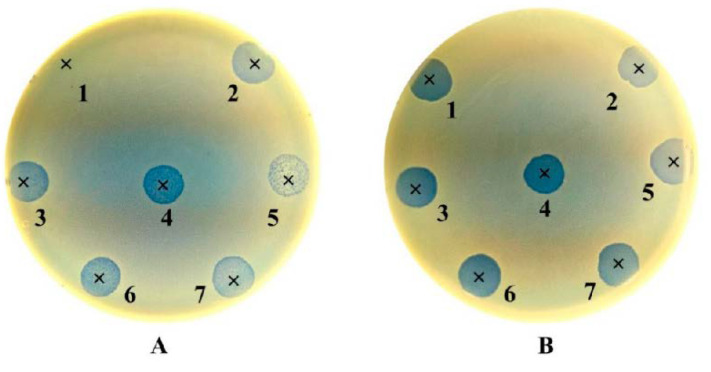
Lytic activity of seven commercial bacteriophage cocktails against *Staphylococcus aureus* reference strain ATCC 15923 before and after bacteriophage adaptation. (1) Staphylococcal Bacteriophage (Eliava); (2) Pyo Bacteriophage (Eliava); (3) Ses Bacteriophage (Eliava); (4) Fersisi Bacteriophage (Eliava); (5) Enko Bacteriophage (Eliava); (6) Intesti Bacteriophage (Eliava); (7) Pyobacteriophag Bacteriophage (Microgen). (**A**) Lytic activity before adaptation procedure. (1) R (−), resistant or no lysis; (2–4) PL (++), partial lysis; (5) IP (+), individual plaques; (6, 7) PL (++), partial lysis. (**B**) Lytic activity after adaptation procedure. (1) CL (+++), confluent lysis; (2) PL (++), partial lysis; (3, 4) CL (+++), confluent lysis; (5) PL (++), partial lysis; (6, 7) CL (+++), confluent lysis.

The visual confirmation results of the effect of the adaptation procedure for exposure of *S. aureus* ATCC 15923 to seven commercial bacteriophage cocktails is shown in Figure 2, which shows that the adaptation procedure led to both improvement in already positive lytic activity and overcoming bacterial resistance.

Staphylococcal Bacteriophage (Eliava) against 11 chosen bacterial strains (9 with resistance and 2 with individual plaques) were taken for phage adaptation. In all cases, the adaptation procedure resulted in overcoming bacterial resistance; all 11 bacterial strains (100%) showed a positive lytic effect after adaptation (see Table 3).

## 4. Discussion

In our study, we detected the clinical relevance of *S. aureus* colonization in PD patients, strain antimicrobial susceptibility, biofilm characteristics, and we performed an in vitro phage susceptibility testing that showed the broad lytic activity of different commercially available bacteriophages indicating a wide phage antimicrobial effect.

The necessity of novel antimicrobial agents that can be used against *S. aureus-*caused infections in PD patients is of great importance, as this microorganism is one of the most prevalent causative agents of PD-associated peritonitis and exit-site infections. Due to its strong virulence profile and biofilm production capability in PD patients, it has high peritonitis relapse, PD catheter removal, and death rates. Our results suggested a 2.33 times greater relative risk of death in the *S. aureus* carrier group that demonstrates worse overall prognosis for *S. aureus* carriers, similarly to other studies. Interestingly, we did not detect higher peritonitis or *S. aureus*-caused peritonitis rate in the carrier group that could be explained by our relatively small study group. Nevertheless, the overall peritonitis rate was 0.35 per patient year, similar to data from other studies having larger cohorts in Northern Europe [40]. 

For PD patients, *S. aureus* colonization is one of the sources of endogenous infection; therefore, use of long-term antimicrobials is recommended by International Society for Peritoneal Dialysis guidelines, but in several countries, it is not fulfilled to avoid possible *S. aureus* resistance development. Our results of *S. aureus* isolation from the nasal cavity, groin region, and PD catheter exit-site indicated 30% (*n* = 21) *S. aureus* colonization among PD patients, which is similar but slightly greater than that in a similar study [41]. Colonization was mainly detected in several body niches, mostly in the nasal cavity (*n* = 18) but also in the groin region (*n* = 11) and around the PD catheter exit site (*n* = 5); however, none of the strains were MRSA.

A positive or lytic bacteriophage effect was detected in all 71 *S. aureus* strains using commercially available bacteriophage cocktails from Eliava (Pyo, Ses, Fersisi, Enko, Intesti) and Microgen (Pyobacteriophag), except Eliava Staphylococcal Bacteriophage, where the incidence of resistance or no lytic effect was 26% (*n* = 9). The most commonly observed degree of positive phage lytic effect was partial lysis, ranging 56–100% among isolates and could depend on phage concentration within the cocktail, time of incubation, and phage resistance. Our results of wide phage lytic activity of 100% for all except Eliava Staphylococcal Bacteriophage could be also associated with possible low strain genetic diversity, as in various parts of the world, we can find genetically and biologically distinct strains of the same bacterial species [42]. In cases of bacterial resistance or a weak lytic effect towards Eliava Staphylococcal Bacteriophage, an adaptation procedure or so-called host range expansion was performed. The outcome after adaptation was indicative of persistent enhancement in bactericidal action, leading to 100% lytic activity. In nine resistant strains, adaptation led to overcoming bacterial resistance; in two strains, improvement of positive lytic activity from individual plaques (+) to partial lysis (++) was achieved. The principle of phage adaptation is mainly due to spontaneous mutations and possible gene recombination between genetically diverse bacteriophages within the phage cocktail. Such changes lead to altered structural gene products, for example, encoding phage tail fiber assembly proteins that are necessary for phage attachment to the host cell or adsorption, which is the first step of phage infection. Adaptation can also reduce lysis time and increase phage burst size [43,44]. Recent findings on the Eliava Staphylococcal Bacteriophage cocktail that consist of Twort-like phage Sb-1 showed that after the adaptation process, newly formed phage clones were found in phage stock, and such a process increased phage lytic activity from 87% to 96% on globally diverse *S. aureus* strains. Interestingly, genetic differences between the mutant and parental Sb-1 phages were found in the phage genome hypervariable complex repeat structure, but the adsorption rate between parental and mutant phage was similar, with the conclusion that the process of host range expansion in the Sb-1 phage is still unclear [30]. Another study from Switzerland showed similar results in that after phage–bacterium adaptation of Eliava Pyo Bacteriophage with *E. coli* isolates from urinary tract infection, the lytic spectrum increased from 65.9% to 92.7% [45]. 

In phage therapy, it is important that the phage is lytic, as lysogenic phages can integrate into the genome of bacteria and introduce new pathogenic properties [46,47]; for example, Shiga toxin genes responsible for *E. coli*-associated hemorrhagic colitis and hemolytic uremic syndrome development that are conveyed by lambdoid lysogenic bacteriophages [48]. In lysogenic phages, these properties can be transferred via horizontal gene transfer; therefore, morphologic characterization of phages and whole-genome sequencing should be performed before therapeutic use [49]. Bacterial resistance towards phages can also emerge, so the use of cocktails containing several phages even against different bacterial species is reasonable. Proper quality and safety procedures are already described by phage researchers in guidelines and articles of phage cocktail preparations [39,50]. Still, the current legal framework of phage therapy for their possible clinical use and easier clinical-study organization is a major hurdle. Already now Eliava bacteriophages have been used to treat life-threatening *S. aureus* caused infections with Pyo, Staphylococcal and Fersisi phage cocktails. Results from these reports show positive clinical outcome and great tolerability when used on skin and mucous membranes [51,52]. Pyo, Eliava phage cocktail and its component ISP phage [35] has been used in clinical trials. In these studies, application of bacteriophages did not elicit any side effect [53,54].

Our results revealed clinically relevant (strong and moderate) biofilm production in 91.2% (*n* = 31) of strains, an observation similar to another study from Brazil where *S. aureus* strains isolated from PD peritonitis patients produced biofilm in 88.7% (*n* = 55) of cases, even though a different biofilm detection method was used [15], indicating that microtiter plate assay can be used as a cost-effective screening method for biofilm detection in clinically relevant *S. aureus* strains. Phage lytic activity was not affected by different degrees of *S. aureus* biofilm production, showing that their biofilm production capability does not interfere with in vitro testing of phage on planktonic bacteria. 

*S. aureus* isolation sites might be of major importance; although the majority of strains were isolated from the nasal cavity, two patients were only colonized in the groin and one in only the PD catheter exit-site regions. Among patients with multiple isolated *S. aureus* strains, their biofilm production phenotype was statistically different (see Figure 1). Based on the study of 130 *S. aureus* isolates, Piechota M. et al. [21] reported, that their biofilm production differed according to their isolation site, and strong biofilm producers were found mainly from tracheostomy tubes, sputum, throat, and nose. We detected strong biofilm production in 56–73% of *S. aureus* among all three isolation sites. The accessory gene regulator (*agr*) quorum-sensing system and intercellular adhesion (*ica*) group genes play an important role in *S. aureus* biofilm formation, but they are environmental factor-dependent [18,19,20,21]. Our results about biofilm production and phage susceptibility support *S. aureus* phenotypic variability, even within one patient; however, such phenomena did not interfere with the bacteriophage positive lytic effect that was detected in the majority of strains.

## 5. Conclusions

Colonization of *S. aureus* was associated with greater mortality rate. In vitro, bacteriophage activity showed a broad lytic spectrum against isolates. In the case of bacterial resistance, it was overcome with an adaptation procedure. Interestingly, the phenotypic differences of *S. aureus* isolates for phage susceptibility and biofilm production were detected, meaning that isolate virulence was variable and isolation site-dependent.

The increasing incidence of antibiotic-resistant infections and the lack of novel antimicrobials against multidrug-resistant bacteria makes phages a promising agent in treating biofilm-associated infections.

## Figures and Tables

**Table 1:** *S. aureus* carrier characteristics. Note: PD, peritoneal dialysis; ADPKD, autosomal dominant polycystic kidney disease.

N	Age	Sex	Cause of End-Stage Renal Disease	Carriage Site (Biofilm Production Group)			Outcome in Two Years
				Nose	Groin	PD Catheter	
1	55	♂	Diabetic nephropathy	1N strong			1 episode of *S. aureus/Pseudomonas* spp. Peritonitis, transplantation
2	74	♀	Glomerulonephritis	3N strong			Death
3	76	♂	Hypertensive nephropathy	6N moderate	6G strong		3 episodes of peritonitis (2 *Streptococcus* spp., 1 culture-negative)
4	83	♂	Chronic interstitial nephritis	8N strong	8G strong		Death
5	68	♀	Diabetic nephropathy	12N moderate		12PD strong	Death
6	36	♂	Chronic interstitial nephritis	14N moderate			Transplantation
7	64	♂	Chronic interstitial nephritis	15N strong	15G strong		Uneventful PD
8	42	♀	Chronic interstitial nephritis	17N strong			Transplantation
9	60	♂	ADPKD	19N moderate			Transplantation
10	34	♂	Glomerulonephritis	25N strong	25G strong	25PD strong	Transplantation
11	77	♀	ADPKD			26PD strong	Death
12	68	♂	Glomerulonephritis	27N strong	27G strong		Uneventful PD
13	69	♂	Glomerulonephritis	35N strong	35G strong		1 episode of culture-negative peritonitis
14	68	♂	Diabetic nephropathy	36N moderate			Transplantation
15	69	♂	Glomerulonephritis		47G strong		Uneventful PD
16	58	♂	Hypertensive nephropathy		48N strong		Death
17	65	♂	Diabetic nephropathy	58N strong			Death
18	81	♂	Chronic interstitial nephritis	59N weak	59G weak		Death
19	26	♀	Glomerulonephritis	67N weak	67G moderate	67PD moderate	2 episodes of peritonitis (1 *Streptococcus* spp., 1 culture-negative)
20	47	♂	Glomerulonephritis	70N strong			Transplantation
21	49	♂	Diabetic nephropathy	71N moderate	71G moderate	71PD moderate	Death
TOTAL *n* (%)				18 (53%)	11 (32%)	5 (15%)	
*S. aureus* Strain Count							
Biofilm Production *n* (%)			Strong	10 (56%)	8 (73%)	3 (60%)	
			Moderate	6 (33%)	2 (18%)	2 (40%)	
			Weak	2 (11%)	1 (9%)		

**Table 2:** Two-year outcome of screened patients.

Clinical Outcomes	*S. aureus* Carriers	*S. aureus*	Total	RR	CI 95%
		Noncarriers			
Number of patients	30.0%	70.0%	100% (*n* = 70)		
	(*n* = 21)	(*n* = 49)			
Death	31.1%	16.3%	22.9% (*n* = 16)	2.33	1.01–5.38
	(*n* = 8)	(*n* = 8)			
Transplantation	28.6%	18.4%	21.4% (*n* = 15)	1.56	0.63–3.81
	(*n* = 6)	(*n* = 9)			
Removal of PD catheter	0%	20.4%	14.3% (*n* = 10)		
	(*n* = 0)	(*n* = 10)			
Peritonitis	19.1%	34.7%	30.0% (*n* = 21)	0.55	0.21–1.44
	(*n* = 4)	(*n* = 17)			

**Table 3:** Lytic activity of seven commercial bacteriophage cocktails against 34 *Staphylococcus aureus* strains acquired from peritoneal dialysis patient samples.

S. aureus Strain Code		Bacteriophages							
	Biofilm Production Degree	Eliava BioPreparations Ltd.						Microgen Ltd.	
		Staphylococcal		Pyo	Ses	Fersisi	Enko	Intesti	Pyobacteriophag
		1.2 × 10^7^		3.4 × 10^7^	4.0 × 10^7^	2.4 × 10^7^	1.4 × 10^7^	2.0 × 10^7^	2.0 × 10^7^
		Not Adapted	Adapted						
1N	Strong	PL		PL	PL	PL	PL	PL	PL
3N	Strong	PL		PL	PL	PL	PL	PL	PL
6N	Moderate	R ^a^	PL	PL	PL	PL	PL	PL	PL
6G	Strong	R ^a^	PL	PL	PL	PL	PL	PL	PL
8N	Strong	PL		PL	PL	PL	PL	PL	PL
8G	Strong	PL		PL	PL	PL	PL	PL	PL
12N	Moderate	IP ^a^	PL	PL	PL	PL	PL	PL	IP
12PD	Strong	R ^a^	PL	PL	PL	PL	PL	PL	PL
14N	Moderate	PL		PL	PL	PL	PL	PL	PL
15N	Strong	R ^a^	PL	PL	PL	PL	PL	PL	PL
15G	Strong	R ^a^	PL	PL	PL	PL	PL	PL	PL
17N	Strong	R ^a^	PL	IP	PL	PL	PL	PL	IP
19N	Moderate	R ^a^	PL	PL	PL	PL	PL	PL	IP
25N	Strong	PL		PL	PL	PL	PL	PL	PL
25PD	Strong	PL		PL	PL	PL	PL	PL	PL
25G	Strong	PL		PL	PL	PL	PL	PL	PL
26PD	Strong	PL		PL	PL	PL	PL	PL	PL
27N	Strong	PL		PL	IP	PL	IP	PL	PL
27G	Strong	PL		CL	PL	PL	CL	PL	PL
35N	Strong	IP ^a^	PL	PL	PL	PL	PL	PL	PL
35G	Strong	PL		PL	PL	PL	PL	PL	PL
36N	Moderate	PL		PL	PL	PL	PL	PL	PL
47G	Strong	CL		PL	PL	PL	PL	PL	PL
48G	Strong	CL		PL	PL	PL	PL	PL	PL
58N	Strong	PL		PL	PL	PL	PL	PL	PL
59G	Weak	CL		PL	IP	PL	PL	PL	PL
59N	Weak	CL		CL	PL	IP	PL	PL	PL
67G	Weak	R ^a^	PL	PL	PL	PL	PL	PL	PL
67N	Moderate	R ^a^	PL	PL	PL	PL	PL	PL	PL
67PD	Moderate	PL		PL	PL	PL	PL	PL	PL
70N	Strong	PL		PL	PL	PL	PL	PL	PL
71PD	Moderate	PL		PL	PL	PL	PL	PL	PL
71G	Moderate	PL		PL	PL	PL	PL	PL	PL
71N	Moderate	PL		PL	PL	PL	PL	PL	PL
	Phage lytic activity	*n* (%)	*n* (%)	*n* (%)	*n* (%)	*n* (%)	*n* (%)	*n* (%)	*n* (%)
	CL (+++)	4 (12)	0 (0)	2 (6)	0 (0)	0 (0)	1 (3)	0 (0)	0 (0)
	PL (++)	19 (56)	11 (100)	31 (91)	32 (94)	33 (97)	32 (94)	34 (100)	31 (91)
	IP (+)	2 ^a^ (6)	0 (0)	1 (3)	2 (6)	1 (3)	1 (3)	0 (0)	3 (9)
	R (−)	9 ^a^ (26)	0 (0)	0 (0)	0 (0)	0 (0)	0 (0)	0 (0)	0 (0)

^a^ Staphylococcal Bacteriophage (Eliava) taken to adaptation procedure after showing spot test result as individual plaques (+) or no lysis (−) against 11 *Staphylococcus aureus* strains acquired from peritoneal dialysis patients samples: CL (+++), confluent lysis; PL (++), partial lysis; IP (+), individual plaques; R (−), resistant or no lysis.

## Supplementary Materials

The following are available online at https://www.mdpi.com/2079-6382/9/9/582/s1, Table S1: Patients characteristics, Table S2: *Staphylococcus aureus* strain biofilm mean optical density and antimicrobial susceptibility, Figure S1: *S. aureus* biofilm growth using microtiter plate assay stained with crystal violet.
