# Supplementary material for: S. aureus Colonization, Biofilm Production, and Phage Susceptibility in Peritoneal Dialysis Patients
Source: Antibiotics (Basel). 2020 Sep 7;9(9):582. doi: 10.3390/antibiotics9090582 (PMC7558627; doi:10.3390/antibiotics9090582)
Supplement: Supplementary file 1 [file antibiotics-09-00582-s001.pdf]

Table S1. Patients characteristics. Note: PD, peritoneal dialysis; ADPKD, autosomal dominant polycystic kidney disease; COPD, chronic obstructive pulmonary disease.

N	Age	Sex	Cause of end-stage renal disease	Outcome in two years	Co-morbidities
1	55	♂	Diabetic nephropathy	1 episode of *S. aureus* / *Pseudomonas spp.* peritonitis, transplantation	Diabetes
2	36	♂	Glomerulonephritis	Transplantation	
3	74	♀	Glomerulonephritis	Death	Diabetes, Chronic heart failure
4	33	♀	Glomerulonephritis	Transplantation	
5	75	♂	Chronic interstitial nephritis	1 episode of methicillin-sensitive *S. aureus* peritonitis	
6	76	♂	Hypertensive nephropathy	3 episodes of peritonitis (2 *Streptococcus spp.*, 1 culture negative)	Chronic heart failure
7	61	♂	Chronic interstitial nephritis	1 episode of *Enterococcus spp.* peritonitis	
8	83	♂	Chronic interstitial nephritis	Death	Chronic heart failure, Gout
9	78	♀	Chronic interstitial nephritis	1 episode of *Streptococcus spp.* peritonitis, death	
10	67	♀	Hypertensive nephropathy	Transplantation	Chronic heart failure
11	56	♂	Chronic interstitial nephritis	Uneventful PD	
12	68	♀	Diabetic nephropathy	Death	Diabetes
13	71	♀	Hypertensive nephropathy	Uneventful PD	Chronic heart failure, COPD
14	36	♂	Chronic interstitial nephritis	Transplantation	Viral hepatitis
15	64	♂	Chronic interstitial nephritis	Uneventful PD	Chronic heart failure
16	69	♀	Diabetic nephropathy	1 episode of *culture negative* peritonitis, removal of PD catheter	Diabetes
17	42	♀	Chronic interstitial nephritis	Transplantation	
18	58	♀	ADPKD	2 episodes of peritonitis (1 *Aeromonas spp., 1 Streptococcus spp.*)	Viral hepatitis
19	60	♂	ADPKD	Transplantation	
20	32	♂	Glomerulonephritis	Uneventful PD	
21	60	♀	ADPKD	Uneventful PD	
22	59	♂	Glomerulonephritis	1 episode of coagulase-negative *Staphylococcus spp.* peritonitis	Chronic heart failure
23	44	♂	Glomerulonephritis	Transplantation	Chronic heart failure
24	64	♀	Unknown	2 episodes of peritonitis (1 *Streptococcus spp.*, 1 culture negative), removal of PD catheter	Chronic heart failure
25	34	♂	Glomerulonephritis	Transplantation	
26	77	♀	ADPKD	Death	
27	68	♂	Glomerulonephritis	Uneventful PD	Chronic heart failure, COPD
28	64	♂	Glomerulonephritis	2 episodes of peritonitis (1 methicllin-sensitive *S. aureus, 1* culture negative), removal of PD catheter	
29	43	♀	Glomerulonephritis	Removal of PD catheter	
30	39	♀	Chronic interstitial nephritis	1 episode of *Streptococcus spp.* peritonitis	

31	52	♂	Diabetic nephropathy	Uneventful PD	Diabetes, Chronic heart failure, COPD
32	79	♀	Diabetic nephropathy	Uneventful PD	Diabetes
33	58	♀	Glomerulonephritis	Death	
34	76	♂	Chronic interstitial nephritis	1 episode of culture negative peritonitis, removal of PD catheter	
35	69	♂	Glomerulonephritis	1 episode of culture negative peritonitis	
36	68	♂	Diabetic nephropathy	Transplantation	Diabetes
37	82	♂	Glomerulonephritis	Uneventful PD	Gout, Viral hepatitis COPD
38	41	♀	Diabetic nephropathy	Death	Diabetes, Viral hepatitis
39	65	♂	Glomerulonephritis	3 episodes of peritonitis (1 methicillin-resistant coagulase-negative *Staphylococcus*, *1 Aerococcus spp./Pseudomonas spp.*, 1 culture negative)	
40	31	♀	Glomerulonephritis	Uneventful PD	
41	68	♀	ADPKD	Removal of PD catheter	
42 *	53	♂	Glomerulonephritis	Uneventful PD	
43	81	♀	Glomerulonephritis	1 episode of *Bacillus spp.* peritonitis	Chronic heart failure, Gout
44	71	♂	Glomerulonephritis	3 episodes of *Streptococcus spp.* peritonitis	Chronic heart failure
45	72	♀	Hypertensive nephropathy	Death	
46	32	♀	Diabetic nephropathy	Death	Diabetes, Gout
47	69	♂	Glomerulonephritis	Uneventful PD	Chronic heart failure, COPD, Viral hepatitis
48	58	♂	Hypertensive nephropathy	Death	
49	60	♀	Glomerulonephritis	Removal of PD catheter	
50	61	♀	ADPKD	Transplantation	
51	58	♂	Glomerulonephritis	Transplantation	
52	36	♀	Chronic interstitial nephritis	1 episode of *Candida spp.* peritonitis	Chronic heart failure
53	87	♀	Hypertensive nephropathy	2 episodes of *Streptococcus spp.* peritonitis	
54	45	♀	Glomerulonephritis	Uneventful PD	Chronic heart failure, COPD
55	72	♂	Chronic interstitial nephritis	Removal of PD catheter	Chronic heart failure
56	77	♀	Glomerulonephritis	Uneventful PD	
57	69	♂	Hypertensive nephropathy	Death	
58	65	♂	Diabetic nephropathy	Death	Diabetes, Chronic heart failure, Gout
59	81	♂	Chronic interstitial nephritis	Death	
60	57	♂	ADPKD	Transplantation	
61	65	♂	Glomerulonephritis	Transplantation	
62	70	♂	Glomerulonephritis	Uneventful PD	
63	79	♀	Hypertensive nephropathy	1 episode of culture negative peritonitis, death	

64	62	♀	Hypertensive nephropathy	1 episode of culture negative peritonitis, removal of PD catheter	
65	77	♂	Unknown	Death	
66	32	♀	Glomerulonephritis	Removal of PD catheter	
67	26	♀	Glomerulonephritis	2 episodes of peritonitis (1 *Streptococcus spp.*, 1 culture negative)	Viral hepatitis
68	70	♂	Chronic interstitial nephritis	Uneventful PD	
69	34	♀	Glomerulonephritis	Transplantation	
70	47	♂	Glomerulonephritis	Transplantation	Chronic heart failure
71	49	♂	Diabetic nephropathy	Death	Diabetes

*Not included-peritoneal dialysis was not initiated.

Table S2. *Staphylococcus aureus* strain biofilm mean optical density and antimicrobial susceptibility. Note: FOX, cefoxitin; CIP, ciprofloxacin; TET, tetracycline; SXT, trimethoprim/sulfamethoxazole; RIF, rifampicin; ERY, erythromycin; CLI, clindamycin; GEN, gentamycin; S, susceptible; R, resistant.

***S. aureus* Strain Code**	**Mean OD at 570 nm**	Antimicrobial susceptibility							
		**FOX**	**CIP**	**TET**	**SXT**	**RIF**	**ERY**	**CLI**	**GEN**
1N	0.23	S	S	S	S	S	S	S	S
3N	0.25	S	S	S	S	S	S	S	S
6N	0.14	S	S	S	S	S	S	S	S
6G	0.48	S	S	S	S	S	S	S	S
8N	0.27	S	S	S	S	S	S	S	S
8G	0.43	S	S	S	S	S	S	S	S
12N	0.16	S	S	S	S	S	S	S	S
12PD	0.29	S	S	S	S	S	S	S	S
14N	0.18	S	S	S	S	S	S	S	S
15N	0.37	S	S	S	S	S	S	S	S
15G	0.52	S	S	S	S	S	S	S	S
17N	0.25	S	S	S	S	S	S	S	S
19N	0.14	S	S	S	S	S	S	S	S
25N	0.42	S	S	S	S	S	S	S	S
25PD	0.32	S	S	S	S	S	S	S	S
25G	0.57	S	S	S	S	S	S	S	S
26PD	0.36	S	S	S	S	S	S	S	S
27N	0.3	S	S	S	S	S	S	S	S
27G	1.04	S	S	S	S	S	S	S	S
35N	0.4	S	S	S	S	S	S	S	S
35G	0.29	S	S	S	S	S	S	S	S
36N	0.19	S	S	S	S	S	S	S	S
47G	0.25	S	S	S	S	S	S	S	S
48G	0.43	S	S	S	S	S	S	S	S
58N	0.36	S	S	S	S	S	S	S	S
59G	0.1	S	S	S	S	S	S	S	S
59N	0.1	S	S	S	S	S	S	S	S
67G	0.21	S	S	S	S	S	R	S	S
67N	0.08	S	S	S	S	S	R	S	S
67PD	0.17	S	S	S	S	S	S	S	S
70N	0.4	S	S	S	S	S	S	S	S
71PD	0.13	S	S	S	S	S	S	S	S
71G	0.13	S	S	S	S	S	S	S	S
71N	0.14	S	S	S	S	S	S	S	S

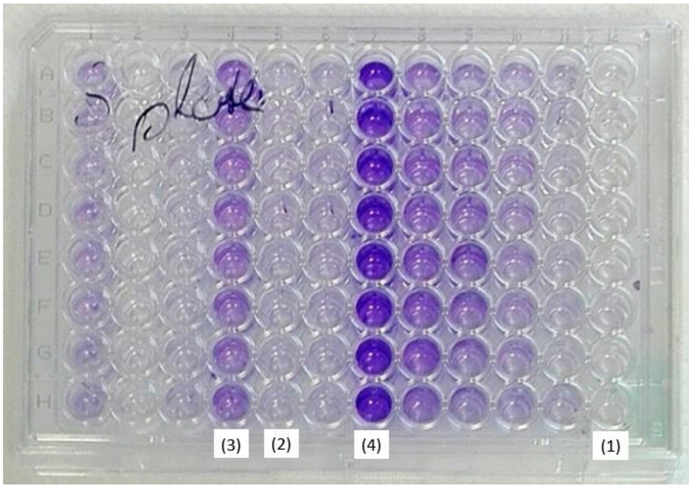

Figure S1. *S. aureus* biofilm growth using microtiter plate assay stained with crystal violet. (1) negative control no biofilm production, (2) weak biofilm producer, (3) moderate biofilm producer, (4) strong biofilm producer.
